# Thrombotic events after vaccination for covid-19 in Italy: a report from the Italian society on thrombosis and haemostasis registry

**DOI:** 10.1007/s11739-025-04054-7

**Published:** 2025-07-30

**Authors:** Marco Paolo Donadini, Elisa Tarasconi, Lorenza Bertù, Emilia Antonucci, Laura Contino, Valerio De Stefano, Rossella Marcucci, Gualtiero Palareti, Laura Russo, Giovanni Luca Tiscia, Armando Tripodi, Paolo Gresele

**Affiliations:** 1 Research Center on Thromboembolic Diseases and Antithrombotic Therapies, Department of Medicine and Surgery, University of Insubria, Medicina d’Urgenza e Centro Trombosi ed Emostasi – ASST Sette Laghi, Via Guicciardini, 9 – 21100 Varese, Italy; 2 SC Medicina Generale II, Ospedale San Paolo, ASST Santi Paolo E Carlo, Università Degli Studi Di Milano, Milan, Italy; 3 Fondazione Arianna Anticoagulazione, Bologna, Italy; 4 Hemostasis and Thrombosis Unit, Translational Medicine Department, University Hospital Alessandria, Alessandria, Italy; 5 Section of Hematology, Department of Radiological and Hematological Sciences, Catholic University, Fondazione Policlinico Gemelli IRCCS, Rome, Italy; 6 Department of Experimental and Clinical Medicine, University of Florence, Florence, Italy; 7 Division of Immunohematology and Transfusion Medicine, Hospital Papa Giovanni XXIII of Bergamo, Bergamo, Italy; 8 UOSD Di Emostasi E Trombosi, Ospedale IRCCS Casa Sollievo Della Sofferenza, San Giovanni Rotondo, Foggia, Italy; 9 Fondazione IRCCS Ca’ Granda Ospedale Maggiore Policlinico, Angelo Bianchi Bonomi Hemophilia and Thrombosis Center, Milan, Italy; 10 Section of Internal and Cardiovascular Medicine, Department of Medicine, University of Perugia, Perugia, Italy

**Keywords:** Vaccine-induced immune thrombotic thrombocytopenia, Thrombosis, Bleeding, Vaccines

## Abstract

Since vaccine induced immune thrombotic thrombocytopenia (VITT) became evident in early 2021, research has focused on characterizing this new pathologic entity. Moreover, data were urgently needed on the association, if any, between all COVID-19 vaccines and thrombotic events (TEs). The study was aimed to collect relevant information on all cases of TEs occurring after COVID-19 vaccination. A national, prospective registry was set up by the Italian Society on Thrombosis and Haemostasis, enrolling consecutive adult patients diagnosed with any TE occurring within 30 days from any COVID-19 vaccine dose, with or without thrombocytopenia. The primary objective was the characterization of TEs and description of its management. The study was approved by the national ethics committee on COVID-19 research and by the participating centers. Among 308 patients included in the registry from February 2021 up to 29 Aug 2022, 276 (89.6%) were diagnosed with venous (266) and/or arterial (33) non-VITT TEs, after a median of 14 days after vaccination. The median age was 60 years, 151 (54.7%) were males, 48 (17.5%) had previous venous or arterial TEs. Within 30 days after diagnosis, 8 (2.9%) patients experienced thrombosis progression/recurrence, 2 (0.7%) major bleeding, 8 (2.9%) died. No differences were found in terms of thrombosis characteristics and progression between non-VITT patients receiving mRNA and adenoviral vector-based vaccines. The remaining 32 patients (10.4%) included in the registry were diagnosed with VITT, after a median of 10 days after vaccination. All of them received adenoviral vector-based vaccines. As compared to non-VITT thrombosis, VITT involved more often both venous and arterial sites (25% vs. 4%, p 0.0002) and unusual sites (62.5% vs. 18.8%, *p* < 0.0001) and was associated with worse outcomes (thrombosis progression/recurrence 25% vs. 2.9%, major bleeding 34.4% vs. 0.7%, death 18.8% vs. 2.9%, for all comparisons *p* < 0.0001). Significant differences were found between non-VITT and VITT cases. Non-VITT thromboses occurring after vaccination seem to resemble common thrombosis phenotypes.

## Introduction

Vaccine induced immune thrombotic thrombocytopenia (VITT) was described as a new pathologic entity in early 2021, as soon as the vaccination campaign against COVID-19 started on a worldwide basis with hundreds of thousands of patients receiving anti-SARS-COV-2 vaccines [1–4]. Since then, research has focused on characterizing VITT both from a pathophysiological and clinical standpoint [5–7]. Moreover, at that timepoint, management and therapeutic strategies were urgently needed and most scientific societies on thrombosis and hemostasis provided guidance based on very preliminary descriptions of pathologic mechanisms and disease clinical course, that resembled heparin-induced thrombocytopenia (HIT) and shared some characteristics with other thrombotic microangiopathies [8–10].

At the same time, it was deemed crucial to collect relevant information on all cases of thrombotic events (TEs) occurring after COVID-19 vaccination and to determine whether an association, if any, existed between all types of COVID-19 vaccines and any type of TE. Those data are still important nowadays, considering that several adenoviral-based vaccines are currently used, and others are undergoing clinical investigation [11, 12], and that additional models of HIT/VITT disease have been recently reported following viral infection [13, 14].

Thus, the Italian Society on Thrombosis and Haemostasis (SISET) set up a national, prospective registry to record and describe all thrombotic events occurring within 30 days after the administration of any COVID-19 vaccine, with or without associated thrombocytopenia, with the aim to explore the clinical characteristics and evaluate clinical management and outcomes in terms of mortality, extension or recurrent thrombosis and bleeding.

## Methods

### Study design and population

The “SISET Vax Covid-19” was a national, multicenter, prospective registry, set up by the Italian Society on Thrombosis and Haemostasis and endorsed by additional Italian scientific societies (see acknowledgments). The registry included consecutive adult patients, aged 18 years or older, diagnosed with any thrombotic event (venous, arterial, microvascular), with or without concomitant thrombocytopenia, occurring within 30 days from any received COVID-19 vaccine dose. Thrombocytopenia was defined as a platelet count less than 100 × 10^9^/L or a 30% platelet count reduction in case of previous thrombocytopenia. The full protocol is available upon request.

Written informed consent was required to participate. No other exclusion criteria were applied.

The registry was set up in June 2021 and was closed on August 31st 2022.

The study was conducted in accordance with the Declaration of Helsinki and was approved by the National Ethics Committee for studies on COVID-19 (approval n. 333-2020/2021 Istituto Nazionale per le Malattie Infettive “L. Spallanzani”, Rome) and by the ethics committees of the participating centers.

### Study outcomes

The primary outcome was the characterization of the thrombotic events occurred after COVID-19 vaccination, in terms of type of vaccination, type and extension of thrombosis, diagnostic methods, therapeutic management and adverse events (thrombosis extension or recurrence, bleeding, mortality) during a 30-day follow-up.

Secondary outcomes included the relative proportions of arterial thrombosis, lower or upper limb deep vein thrombosis, venous thrombosis at unusual site (e.g., cerebral venous thrombosis, splanchnic vein thrombosis), major or clinically relevant non-major bleeding, thrombocytopenia, thrombotic microangiopathies (TMA) and disseminated intravascular coagulation (DIC).

Major bleeding and clinically relevant non-major bleeding were defined following the criteria of the International Society on Thrombosis and Haemostasis. Accordingly, bleeding is defined as major if it is fatal and/or if it involves critical areas or organ such as intracranial, intraspinal, intraocular, intra-articular, retroperitoneal, pericardial or intramuscular with compartment syndrome and/or if it causes a decrease in hemoglobin concentration of 2.0 g/dL or more or if it leads to transfusion of two or more units of whole blood or red cells [15]. Clinically relevant non-major bleeding is bleeding which does not fit the criteria for major bleeding but requires medical intervention by a healthcare professional or leads to hospitalization or prompts a face-to-face evaluation [16].

### Data collection

At study entry, the following information was collected for each patient: demographic characteristics, such as age, gender, weight and height; medical history, including previous thrombotic events and known risk factors for thrombosis; characteristics of the index event, including site, nature and extension of thrombosis; laboratory and diagnostic tests; management, dosage and duration of anticoagulants and other drugs administered to treat VITT and adverse events.

All data were collected in electronic form, through the REDCap platform. Fondazione Arianna Anticoagulazione provided support for web-based dataset implementation and data management.

Data were entered anonymously, and each enrolled patient was uniquely identified by a progressive entry number.

Fondazione Arianna Anticoagulazione, along with the coordinating centers of Perugia and Varese, monitored and reviewed data and, in case of missing information or inconsistencies, sent queries to local investigators.

All cases of VITT were adjudicated independently by three blinded experts (VDS, PG, RM), according to the criteria proposed by Pavord et al. [17]. Cases where there was no unanimous agreement were examined in a second round of discussion with an open debate. In all cases unanimity was reached.

A formal sample size was not calculated due to the exploratory nature of the study on a new disease and the absence of information from previous studies.

### Data analysis

All the variables collected in the SISET-Vax Covid-19 database were summarized using descriptive statistical measures. Statistical analyses were carried out using the SAS v9.4 software.

For discrete variables, absolute and percentage frequencies were calculated, while for continuous variables, mean, standard deviation (SD), median and minimum and maximum values were used.

Post-hoc analysis was conducted, comparing patients diagnosed with VITT and without VITT.

Furthermore, among the latter group, patients vaccinated with Adenoviral vaccine and those with mRNA vaccine were compared. Comparisons between groups were conducted using the chi-square test for categorical variables and the Wilcoxon Mann–Whitney for continuos variables.

## Results

Among 308 patients included in the registry over a 15 months period, from 45 italian centers, 276 (89.6%) were diagnosed with venous (266 patients, 96.4%) and/or arterial (33 patients, 12%) non-VITT thrombotic events, after a median of 14 (range 0–35) days after vaccination. The median age was 60 (range 18–97) years, 151 (54.7%) were males, 48 (17.5%) had previous venous or arterial TEs. Within 30 days after diagnosis, 8 (2.9%) patients experienced thrombosis progression/recurrence, 2 (0.7%) had major bleeding and 8 (2.9%) died (Table 1).

**Table 1:** Baseline characteristics and outcomes

	VITT	Non VITT	*P*-value
Patients, *n*	32	276	
Age, years, median (range)	58 (20–77)	60 (18–97)	0.69
Males/Females, *n* (%)	11 (35.5)/20(64.5)	151 (54.7)/125 (45.3)	0.04
Previous COVID infection yes/no (%)	31 (96.9)/1 (3.1)	229 (83.0)/33 (11.9)	0.15
Previous VTE, *n* (%)	1 (3.1)	48 (17.5)	0.04
Vaccine type, *n* (%)			< 0.0001
ChADOx1nCOV-19 (Astrazeneca)	27 (84.4)	57 (21.4)	
Ad26.COV2.S (Janssen)	5 (15.6)	2 (0.8)	
BNT162b2 (Pfizer/BioNTech)	0 (0.0)	167 (62.8)	
mRNA-1273 (Moderna)	- 0 (0.0)	- 40 (15.0)	
Time interval between vaccine and thrombosis, days, median (range)	10 (5–23)	14 (0–35)	0.32
1 st dose / subsequent dose, *n* (%)	31 (96.9)/1 (3.1)	145 (52.5)/131 (47.5)	< 0.0001
Thrombocytopenia, *n* (%)*	32 (100)	18 (6.5)	< 0.0001
Thrombosis site in patients, *n* (%)			
Venous thrombosis	28 (87.5)	266 (96.4)	0.02
Lower or upper limb DVT and/or PE	8 (25.0)	233 (84.4)	< 0.0001
Unusual site venous thrombosis	20 (62.5)	33 (18.8)	< 0.0001
Arterial thrombosis	12 (37.5)	33 (12.0)	< 0.0001
Concomitant arterial and venous thrombosis	8 (25.0)	11 (4.0)	0.0002
Hospitalization yes/no (%)	30 (96.9)/1 (3.1)	152 (55.1)/124 (44.9)	< 0.0001
Thrombosis progression, *n* (%)	8 (25.0)	8 (2.9)	< 0.0001
Bleeding *n* (%)			
Major bleeding	9 (28.1)	2 (0.7)	< 0.0001
Clinically relevant non-major bleeding	1 (3.1)	6 (2.2)	0.54
Death, *n* (%)	6§ (18.8)	8 (2.9)	0.001

*VITT* Vaccine induced immune thrombotic thrombocytopenia, *DVT* deep vein thrombosis, *PE* pulmonary embolism, *SD* standard deviation, *n.a.* not applicable

*Data available on 274 patients

**Thrombosis sites can be multiple

^§^All cases of death among VITT patients, as compared to 4 of 8 among patients without VITT, occurred during the first semester of 2021 (*p* 0.08)

Among non-VITT patients, thrombotic events occurred more frequently after the first/single dose vs. subsequent dose(s) of adenoviral vector-based vaccines as compared to mRNA-based vaccines (70.1% vs. 48.1%, *p* 0.003). No differences were found in terms of thrombosis characteristics and progression between non-VITT patients receiving mRNA and adenoviral vector-based vaccines, whereas clinically relevant bleeding events occurred more frequently after adenoviral vector- based vaccines (8.3% vs. 1.4%, *p* 0.015) (Table 2).

**Table 2:** Baseline characteristics and outcomes of non-VITT patients receiving adenoviral vector- and mRNA-based vaccines

	ChADOx1nCOV-19 (Astrazeneca) or (Ad26.COV2.S) Janssen latest vaccine dose	BNT162b2 (Pfizer/BioNTech) or mRNA-1273 (Modern)a latest vaccine dose	P-value
Patients, n*	60	210	
Age, years, median (range)	65 (21–80)	59 (18–97)	0.15
Males/Females, *n* (%)	33 (55.0)/27 (45.0)	116 (55.2)/94 (44.8)	0.97
Time interval between vaccine and thrombosis, days, median (range)	14 (0–35)	13 (0–35)	0.35
1 st dose / subsequent dose, *n* (%)	42 (70.0)/18 (30.0)	101 (48.1)/109 (51.9)	0.003
Thrombocytopenia, *n* (%)	4 (6.7)	14 (6.7)	0.99
Thrombosis site, *n* (%)**			
Venous thrombosis	55 (91.7)	194 (92.4)	0.79
Lower or upper limb DVT and/or PE	49 (81.7)	171(81.4)	0.99
Unusual site venous thrombosis	6 (10.0)	23 (10.0)	0.99
Arterial thrombosis	10 (16.7)	20 (9.5)	0.16
Concomitant arterial and venous thrombosis	5 (8.3)	5 (2.4)	0.05
Thrombosis progression, *n* (%)	1 (1.7)	7 (3.3)	0.69
Bleeding *n* (%)			
Major bleeding	2 (3.3)	0 (0.0)	0.05
Clinically relevant non-major bleeding	3 (5.0)	3 (1.4)	0.01
Death, *n* (%)	2 (3.3)	6 (2.9)	0.99

*BMI* body mass index, *DVT* deep vein thrombosis, *PE* pulmonary embolism, *SD* standard deviation, *n.a.* not applicable

*Data available on 278 out of 284 patients

**Thrombosis sites can be multiple

The remaining 32 patients (10.4%) included in the registry were diagnosed with VITT, occurring after a median of 10 (range 5–23) days after vaccination with adenoviral-based vaccine (27 patients received ChADOx1 nCOV-19, 5 patients Ad26.COV2.S). These patients showed a mean platelet count at diagnosis of 59 × 10^9^/L (SD 40.1) and at nadir of 45 × 10^9^/L (SD 37.2). D-dimer was available for 28 patients and it was > 4000 ng/mL in 27 (96.4%). Immunoassay for anti PFA/heparin antibodies was performed in 24 patients and it was positive in 19 (67.9%), whereas a positive platelet functional assay was positive in all 6 patients who were tested (100%). VITT was classified as definite in 17 patients, probable in 9 and possible in 6 (Table 3).

**Table 3:** VITT patients’ clinical characteristics

ID#	Age	Male/female	Vaccine type	Dose	Time interval between vaccine and thrombosis, days
3a. Baseline characteristics					
341–6	61	Male	ChADOx1 nCOV-19	1st	15
349–1	60	Male	ChADOx1 nCOV-19	1st	13
350–1	49	Male	ChADOx1 nCOV-19	1st	10
350–2	33	Male	ChADOx1 nCOV-19	2nd	10
353–1	68	Male	Ad26.COV2.S	1st	15
357–12	62	Female	ChADOx1 nCOV-19	1st	5
357–5	52	Male	ChADOx1 nCOV-19	1st	24
357–6	56	Female	ChADOx1 nCOV-19	1st	0
360–1	75	Female	ChADOx1 nCOV-19	1st	10
360–2	55	Female	ChADOx1 nCOV-19	1st	15
363–5	64	Female	ChADOx1 nCOV-19	1st	14
364–3	70	Female	ChADOx1 nCOV-19	1st	8
370–1	60	Female	ChADOx1 nCOV-19	1st	16
370–2	42	Female	ChADOx1 nCOV-19	1st	9
370–4	74	Female	ChADOx1 nCOV-19	1st	20
370–5	57	Female	ChADOx1 nCOV-19	1st	12
370–8	76	Female	ChADOx1 nCOV-19	1st	10
370–10	78	Unknown	Ad26.COV2.S	1st	18
372–2	65	Female	Ad26.COV2.S	1st	20
373–1	46	Male	ChADOx1 nCOV-19	1st	8
376–1	27	Female	ChADOx1 nCOV-19	1st	14
376–2	77	Female	ChADOx1 nCOV-19	1st	15
380–1	41	Female	ChADOx1 nCOV-19	1st	10
381–1	50	Male	ChADOx1 nCOV-19	1st	9
381–2	37	Female	ChADOx1 nCOV-19	1st	19
382–8	20	Female	ChADOx1 nCOV-19	2nd	16
383–1	65	Female	ChADOx1 nCOV-19	1st	10
385–1	52	Female	ChADOx1 nCOV-19	1st	9
385–2	68	Male	ChADOx1 nCOV-19	1st	18
389–1	79	Female	Ad26.COV2.S	1st	19
402–3	44	Male	Ad26.COV2.S	1st	14
399–2	72	Male	ChADOx1 nCOV-19	1st	8

ID#	Venous thrombosis	Venous thrombosis site	Arterial thrombosis	Arterial thrombosis site	Bleeding at diagnosis
3b Type of clinical event at diagnosis					
341–6	Yes	Pulmonary embolism Splanchnic vein thrombosis (portal, mesenteric and splenic vein)	Yes	Splenic artery thrombosis	No
349–1	Yes	Splanchnic vein thrombosis (portal and mesenteric vein)	No	–	No
350–1	Yes	Cerebral vein thrombosis (superior sagittal sinus, transverse sinus, right sigmoid sinus)	No	–	Major bleeding; Intracerebral hemorrhage
350–2	Yes	Pulmonary embolism Cerebral vein thrombosis (superior sagittal sinus, straight sinus, right transverse sinus, right internal jugular vein)	Yes	Thrombosis of abdominal aorta	No
353–1	Yes	Proximal lower limb deep vein thrombosis Pulmonary embolism Splanchnic vein thrombosis (portal vein) Inferior vena cava thrombosis	No	–	No
357–12	No	–	Yes	Superior mesenteric artery, splenic artery, and common hepatic artery thrombosis	No
357–5	Yes	Cerebral vein thrombosis	No	–	Major bleeding; Intracerebral hemorrhage
357–6	Yes	Cerebral vein thrombosis	No	–	No
360–1	Yes	Pulmonary embolism Cerebral vein thrombosis (right internal jugular vein, transverse sinus, sigmoid sinus)	Yes	Stroke	No
360–2	Yes	Pulmonary embolism	No	–	No
363–5	Yes	Pulmonary embolism Cerebral vein thrombosis (sigmoid sinus)	No	–	No
364–3	No	–	Yes	Myocardial infarction	No
370–1	Yes	Pulmonary embolism Proximal lower limb deep vein thrombosis Cerebral vein thrombosis (left cavernous sinus) Splanchnic vein thrombosis (portal vein) Left renal vein thrombosis	No	–	No
370–2	No	–	Yes	Stroke	No
370–4	Yes	Pulmonary embolism Cerebral vein thrombosis (left transverse sinus, left sigmoid sinus) Left renal vein thrombosis	Yes	Stroke Right lower limb peripheral ischemia	No
370–5	Yes	Pulmonary embolism Splanchnic vein thrombosis (portal vein)	Yes	Thrombosis of abdominal aorta Splenic artery thrombosis	No
370–8	Yes	Cerebral vein thrombosis (left transverse sinus, left sigmoid sinus) Splanchnic vein thrombosis (portal vein)	No	–	No
370–10	Yes	Proximal lower limb deep vein thrombosis Distal lower limb deep vein thrombosis Lower limb superficial vein thrombosis	No	–	No
372–2	Yes	Distal lower limb deep vein thrombosis	no	–	No
373–1	Yes	Splanchnic vein thrombosis (portal and splenic vein)	Yes	Myocardial infarction	No
376–1	Yes	Cerebral vein thrombosis (Inferior sagittal sinus, straight sinus, sigmoid sinus)	no	–	Major bleeding; Intracerebral hemorrhage
376–2	No	–	Yes	Bilateral iliac thrombosis	No
380–1	Yes	Pulmonary embolism Cerebral vein thrombosis (straight sinus, sigmoid sinus, transverse sinus) Splanchnic vein thrombosis (portal vein)	no	–	No
381–1	Yes	Pulmonary embolism	no	–	No
381–2	Yes	Splanchnic vein thrombosis (portal, mesenteric and splenic vein)	no	–	No
382–8	Yes	Cerebral vein thrombosis (Superior sagittal sinus	no	–	No
383–1	Yes	Splanchnic vein thrombosis (portal vein)	Yes	Bowel ischemia	No
385–1	Yes	Cerebral vein thrombosis	No	–	Major bleeding; Intracerebral hemorrhage
385–2	Yes	Splanchnic vein thrombosis (portal, mesenteric and splenic vein)		Bowel ischemia	No
389–1	Yes	Distal lower limb deep vein thrombosis	no	–	No
402–3	Yes	Splanchnic vein thrombosis (right suprahepatic vein) Left renal vein thrombosis	no	–	No
399–2	Yes	Inferior vena cava thrombosis	no	–	No

ID#	Platelet count at diagnosis (n/mm3)	Platelet count nadir (n/mm3)	Platelet count at hospital discharge (n/mm3)	Immunoassay (antiPFA/heparin antibodies)	Heparin-induced Platelet Activation (HIPA)	Serotonin release assay (SRA)	D-Dimer (ng/mL)	VITT classification
3c Laboratory tests and VITT classification								
341–6	85,000	50,000	120,000	Positive	Positive	Not performed	> 9000	Definite
349–1	15,000	15.000	253,000	Positive	Not performed	Positive	4309	Definite
350–1	38,000	33,000	323,000	Positive	Positive	Non performed	66,331	Definite
350–2	26,000	25,000	160,000	Positive	Not performed	Not performed	70,374	Definite
353–1	7000	7000	230,000	Positive	Not performed	Not performed	32,533	Definite
357–12	50,000	40,000	90,000	Not performed	Not performed	Not performed	Not performed	Possible
357–5	60,000	20,000	90,000	Not performed	Not performed	Not performed	Not performed	Possible
357–6	60,000	n/a	n/a	Not performed	Not performed	Not performed	Not performed	Possible
360–1	59,000	39,000	289,000	Negative	Not performed	Not performed	63,690	Probable
360–2	137,000	137,000	267,000	Negative	Not performed	Not performed	14,625	Probable
363–5	23,000	23,000	151,000	Not performed	Not performed	Not performed	2500	Possible
364–3	47,000	30,000	428,000	Not performed	Not performed	Not performed	Not performed	Possible
370–1	69,000	24,000	313,000	Positive	Not performed	Not performed	20,782	Definite
370–2	70,000	49,000	385,000	Positive	Not performed	Not performed	70.000	Definite
370–4	95,000	95,000	224,000	Positive	Not performed	Not performed	5151	Definite
370–5	10,000	10,000	157,000	Not performed	Not performed	Not performed	7690	Probable
370–8	23,000	23,000	100,000	Positive	Not performed	Not performed	53,800	Definite
370–10	24,000	24,000	200,000	Positive	Positive	Not performed	87,037	Definite
372–2	91,000	80,000	314,000	Not performed	Not performed	Not performed	4647	Probable
373–1	55,000	55,000	180,000	Negative	Negative	Not performed	4250	Probable
376–1	134,000	130,000	234,000	Positive	Not performed	Not performed	12,204	Definite
376–2	13,000	13,000	210,000	Positive	Not performed	Positive	6863	Definite
380–1	42,000	27,000	250,000	Positive	Not performed	Not performed	18,272	Definite
381–1	42,000	22,000	170,000	Positive	Not performed	Not performed	7600	Definite
381–2	123,000	106,000	178,000	Positive	Not performed	Not performed	3400	Probable
382–8	139,000	125,000	230,000	Positive	Not performed	Not performed	8918	Definite
383–1	45,000	44,000	224,000	Positive	Positive	Not performed	35,000	Definite
385–1	50,000	14,000	n/a	Not performed	Not performed	Not performed	35,200	Probable
385–2	n/a	27,000	n/a	Negative	Not performed	Not performed	34,551	Probable
389–1	24,000	24,000	119,000	Positive	Positive	Not performed	87,037	Definite
402–3	38,000	38,000	90,000	Negative	Not performed	Not performed	39,353	Probable
399–2	134,000	n/a	n/a	Negative	Not performed	Not performed	20,557	Possible

ID#	Anticoagulant	Antiplatelet	Corticosteroids	I.v. Immunoglobulin	Plasma exchange	Other
3d Treatment						
341–6	Fondaparinux	No	Prednisone 25 mg	Yes, 1 g /Kg	No	
349–1	Fondaparinux	No	Dexamethasone 40 mg	Yes, 1 g /kg	Yes	Platelet transfusion
350–1	Fondaparinux	No	Methylprednisolone 20 mg, Dexamethasone 40 mg	Yes, 1 g /kg	No	Lacosamide 200 mg
350–2	Fondaparinux—Argatroban. Apixaban	ASA 100 mg	Dexamethasone 40 mg	Yes, 1 g /kg	No	No
353–1	Fondaparinux, Apixaban	No	Methylprednisolone 80 mg	Yes, 1 g /kg	No	No
357–12	Enoxaparin	No	No	Yes, unknown dosage	No	No
357–5	Enoxaparin	No	No	Yes, unknown dosage	No	No
357–6	Fondaparinux	No	No	Yes, unknown dosage	Yes	No
360–1	Fondaparinux, Apixaban	No	Dexamethasone 40 mg	1 g /kg	No	No
360–2	Fondaparinux, Edoxaban	No	No	No	No	No
363–5	Fondaparinux	No	Prednisone, unknown dosage	Yes, unknown dosage	No	No
364–3	Bivalirudin	Ticagrelor, Cangrelor; ASA 100 mg	Methylprednisolone 40 mg, then 1 mg/kg/die	Yes, 1 g/kg/die	No	No
370–1	Fondaparinux	No	Dexamethasone, unknown dosage	Yes, unknown dosage	No	No
370–2	Fondaparinux	No	Dexamethasone 40 mg	Yes, 1 g/kg	No	No
370–4	Fondaparinux, Warfarin	No	Methylprednisolone 20 mg	No	No	No
370–5	Fondaparinux	ASA 100 mg	Dexamethasone 40 mg	Yes, 1 g/kg	No	No
370–8	Fondaparinux	No	Dexamethasone 12 mg	Yes, 1 g/kg	No	Plasma transfusion
370–10	Fondaparinux	No	Dexamethasone 16 mg	Yes, 1 g/kg	No	Plasma transfusion
372–2	Fondaparinux	No	Prednisone 25	No	No	No
373–1	Enoxaparin Fondaparinux	No	Dexamethasone 40 mg	No	No	No
376–1	Argatroban Fondaparinux Warfarin	No	Dexamethasone 40 mg	Yes, 1 g/kg	No	Levetiracetam
376–2	Argatroban	No	Dexamethasone 40 mg	Yes, 1 g/kg	Yes	No
380–1	Fondaparinux	No	Methylprednisolone 60 mg	Yes, 3000 mg	No	No
381–1	Enoxaparin, Fondaparinux Edoxaban	No	No	Yes, 2 gr/kg	No	No
381–2	Enoxaparin, Agatroban Fondaparinux Apixaban	No	No	Yes, 2 gr/kg	No	No
382–8	Fondaparinux Dabigatran	No	No	Yes, 2 g	No	No
383–1	Fondaparinux, Warfarin	No	Methylprednisolone 60 mg	Yes, 30 g	No	No
385–1	No	No	No	no	No	Ventriculostomy
385–2	Argatroban	No	Dexamethasone 40 mg	Yes, 1 g /kg	No	Platelet transfusion
389–1	Fondaparinux Apixaban	No	Dexamethasone 16 mg	Yes, 1 g /kg	No	Plasma transfusion
402–3	Fondaparinux	No	Methylprednisolone 40 mg	No	No	No
399–2	Fondaparinux Rivaroxaban	No	No	No	No	No

ID#	Thrombosis progression/ recurrence	Bleeding expansion or new event (classification)	Bleeding site	Death
3e. Follow-up				
341–6	No	No		Yes
349–1	No	No		No
350–1	No	No		No
350–2	No	No		No
353–1	No	No		No
357–12	Yes	No		No
357–5	No	No		Yes
357–6	No	Yes (major)	Intracerebral	Yes
360–1	No	Yes (major)	Intracerebral	No
360–2	No	No		No
363–5	No	No		No
364–3	Yes	No		No
370–1	No	No		No
370–2	Yes	Yes (major)	Intracerebral	No
370–4	No	No		No
370–5	Yes	No		No
370–8	No	Yes (major)	Intracerebral and subdural	Yes
370–10	No	No		No
372–2	No	No		No
373–1	Yes	No	-	No
376–1	No	Yes (major)	Intracerebral	No
376–2	No	No		No
380–1	Yes	Yes (major)	Intracranial	No
381–1	No	No		No
381–2	No	Yes (major)	Gastrointestinal	No
382–8	No	No	-	No
383–1	No	Yes (CRNMB)	Gastrointestinal	No
385–1	Yes	Yes (major)	Intracerebral	Yes
385–2	Yes	Yes (major)	Gastrointestinal	Yes
389–1	No	No		No
402–3	No	No		No
399–2	No	No		No

*CRNMB* Clinically relevant non major bleeding

PF4 platelet-factor 4

Patients with VITT, as compared to those with TEs not classified as VITT, were more frequently females (64.5% vs. 45.3%, *p* 0.04), less likely showed previous VTE (3.1% vs. 17.5%, *p* 0.04) and, among vaccine doses, received more often the first one (96.9% vs. 52.5%, *p* < 0.0001). Moreover, VITT was always associated with thrombocytopenia (100% vs. 6.5%, *p* < 0.0001), involved more often both venous and arterial sites (25% vs. 4%, *p* 0.0002), unusual sites (62.5% vs. 18.8%, *p* < 0.0001) and was associated with worse outcomes, including thrombosis progression/recurrence (25% vs. 2.9%, *p* < 0.0001), major bleeding (34.4% vs. 0.7%, *p* < 0.0001) and death (18.8% vs. 2.9%, *p* < 0.0001). (Table 1).

Full description of patients with VITT is provided in Table 3a–e.

## Conclusions

The SISET-VAX COVID-19 registry included 308 patients from 45 centers across Italy who received vaccination against COVID-19 and were diagnosed with a thrombotic event within 30 days. Among them 32 patients (10.4%) developed VITT, which confirmed to be a peculiar thrombotic disease characterized by severe clinical presentation with frequent involvement of both venous and arterial sites (25%) and of unusual locations (62.5%). Moreover, major bleeding complicating the clinical scenario in about one third of cases at presentation and/or in the following few days and mortality was significant (18.8%). These results confirm those described in a previous case series and meta-analysis [5].

On the other hand, thrombotic events not classified as VITT seem to resemble typical phenotypes of VTE in terms of demographic characteristics (age at presentation, gender distribution), clinical presentation (typical venous site involvement) and outcomes, as described in a previous VTE registry on a geographically similar population [18]. It should also be acknowledged that these registries may overrepresent more severe or complex cases, possibly limiting the generalizability of the findings to broader clinical settings. Although the SISET registry was not designed to capture incidence rates—due to the lack of a defined denominator—the number and characteristics of observed non-VITT thrombotic events appear broadly consistent with background rates in the general population, as reported in pre-pandemic and post-vaccination surveillance studies [19, 20]. These findings are also in line with published Italian data estimating the annual incidence of VTE in the general adult population to range between 50 and 100 cases per 100,000 person-years [21].

Indeed, when comparing patients diagnosed with VITT to those with non-VITT thrombotic events in our study population, differences in terms of clinical presentation and outcomes were found to be statistically significant. Patients without VITT generally had a higher risk of thrombosis due to a higher proportion of previous thrombotic events. Despite the increased risk, patients without VITT experienced a lower rate of progression of blood clots than those with VITT, confirming that the latter group had a more severe clinical presentation. Therefore, thrombotic events temporarily occurring after vaccination are clearly different whether they are VITT- or non-VITT-associated thrombosis.

Moreover, after excluding VITT cases, there was no difference in terms of thrombosis characteristics and progression between patients receiving mRNA or adenoviral vector-based vaccines, pointing against a causative role of certain type of vaccines on the occurrence of typical VTE [22] and suggesting these events likely reflect baseline thrombotic risk factors rather than vaccine-specific effects. Individual predispositions such as prior thrombosis, cancer, or immobility may be more relevant triggers in this setting. These results agree with larger epidemiologic evidence, that excluded a causal relationship between m-RNA-based vaccines and thrombotic events [23, 24].

Our study has some limitations. First, the study was intended to prospectively enroll patients starting from the very early description of VITT cases in March 2021. However, the timing of approval from the national ethics committee delayed the study start up to the end of May 2021. Therefore, an amendment to the protocol was made afterward, aimed to include patients retrospectively from March to May 2021. For this reason, for some patients diagnosed with thrombotic events during that period, data were not complete. To address this, participating centers were asked to retrieve as much information as possible from all the available electronic database and paper-based clinical documentation to obtain the most accurate available information. Data entries were subsequently reviewed by the coordinating centers and the data management team, and queries were issued to resolve inconsistencies or fill in missing values where feasible. Nonetheless, some residual missing or imprecise data may remain, which represents a limitation for analyses involving early cases. Second, the sample size was not estimated a priori due to the explorative nature of the registry, on a completely new pathologic entity with lack of preliminary data. However, the registry proposed by SISET was endorsed by several Italian scientific societies and tried to include as many centers as possible across the country to collect a wide range of information on these patients. Third, the study does not include a control group, thus preventing a direct comparison with patients diagnosed with thrombotic events who did not receive vaccination against Covid-19 in the previous 30 days, therefore limiting also causal inference and the ability to estimate relative risks. However, this objective was beyond the aim of the study that was designed to extensively characterize the thrombotic events occurring after vaccination, to collect data on the very rare VITT cases, to verify their possible occurrence after any type of COVID-vaccines and to describe the thrombotic events with or without thrombocytopenia. Finally, regarding VITT, the risk of misclassification cannot be excluded, particularly for cases classified as “probable” or “possible.” This limitation stems from the fact that platelet functional assays—essential for diagnostic confirmation—were performed in only a minority of cases, due to their limited availability, technical complexity, and the need for specialized expertise found in only a few laboratories focused on platelet pathophysiology [25]. Nevertheless, all cases were independently adjudicated by three experts, who were blinded to each other’s assessments and reached consensus after a second round of open discussion in cases of initial disagreement.

In conclusion, the study provided a deep characterization of thrombotic events occurring after vaccination against CODIV-19, concluding that, except for the very rare VITT cases, the clinical characteristics and outcomes of the thrombotic events are comparable to what is known from the literature on typical thrombotic events, thus supporting the epidemiologic evidence of lack of association between thrombotic events and mRNA-based vaccines. Moreover, no significant differences were found between non-VITT thrombotic events diagnosed in patients who received RNA vs. adenoviral-based vaccines.

The study confirms also that VITT is a very rare complication of vaccination with adeno-viral based vaccines and that it is substantially different from common VTE and arterial thrombotic events in terms of pathophysiology, clinical characteristics, and outcomes. It should be emphasized that although VITT is relatively rare, it is associated with high morbidity and mortality and should therefore be always considered when a patient is diagnosed with thrombosis, especially if occurring at unusual sites, after receiving an adeno-viral based vaccine within 30 days.

Further studies are necessary to increase understanding of the pathophysiology and clinical characteristics of VITT, to inform on the best diagnostic and therapeutic strategies. Indeed, studies should explore the mechanisms triggering anti-PF4 antibody formation after vaccination, potential genetic or immunologic predispositions, and differences between VITT and HIT. At the same time, except from this very rare disease, other thrombotic events occurring after vaccines should not be considered a specific type of thrombosis based on epidemiologic study and on the clinical feature of the events, and a causative role of vaccine should be excluded.

## Data Availability

The data supporting the findings of this study are available from the corresponding author upon reasonable request and after notification to and approval from the SISET Executive Board.
